# Severe Rhabdomyolysis without Systemic Involvement: A Rare Case of Idiopathic Eosinophilic Polymyositis

**DOI:** 10.1155/2015/908109

**Published:** 2015-07-01

**Authors:** Ayesha Farooq, Vivek Choksi, Andrew Chu, Dhruti Mankodi, Sameer Shaharyar, Keith O'Brien, Uday Shankar

**Affiliations:** Aventura Hospital and Medical Center Internal Medicine Department, Aventura, FL 33180, USA

## Abstract

*Introduction*. Eosinophilic polymyositis (EPM) is a rare cause of rhabdomyolysis characterized by eosinophilic infiltrates in the muscle. We describe the case of a young patient with eosinophilic polymyositis causing isolated severe rhabdomyolysis without systemic involvement. *Case Presentation*. A 22-year-old Haitian female with no past medical history presented with progressive generalized muscle aches without precipitating factors. Examination of the extremities revealed diffuse muscle tenderness. Laboratory findings demonstrated peripheral eosinophilia and high creatinine phosphokinase (CPK) and transaminase levels. Workup for the common causes of rhabdomyolysis were negative. Her CPK continued to rise to greater than 100,000 units/L so a muscle biopsy was performed which showed widespread eosinophilic infiltrate consistent with eosinophilic polymyositis. She was started on high dose systemic corticosteroids with improvement of her symptoms, eosinophilia, and CPK level. *Discussion*. This case illustrates a systematic workup of rhabdomyolysis in the presence of peripheral eosinophilia. Many differential diagnoses must be considered before establishing a diagnosis of idiopathic eosinophilic polymyositis. To our knowledge, our case of eosinophilic polymyositis is unique as it presented with severe rhabdomyolysis without another organ involvement. Clinicians should maintain a high index of suspicion for this physically debilitating disease to aid in prompt diagnosis.

## 1. Introduction

Rhabdomyolysis is a common condition with multiple causes including physical exertion, trauma, and inflammation. Among the less common etiologies of this finding is eosinophilic polymyositis (EPM). Eosinophilic polymyositis is a rare disease with only a handful of cases [1–16] reported in the literature, mostly in the setting of malignancy [11], autoimmune disease [5], genetic abnormalities [8], hypereosinophilic syndrome (HES) [17], and even medications [1, 4, 7]. When no etiologic factor can be identified, idiopathic eosinophilic polymyositis is diagnosed. It appears that only two cases of idiopathic eosinophilic polymyositis without systemic involvement have been reported [2, 10]. We present the rare case of a young woman with idiopathic eosinophilic polymyositis presenting with muscle pain without involvement of other organ systems. This will be followed by a discussion of the approach to this condition, from clinical presentation to therapy.

## 2. Case Presentation

A 22-year-old Haitian female with no significant prior history presented to the emergency department with severe and generalized muscle aches for the past week mainly involving the shoulder and thigh muscles. Two days prior to presentation, her muscle aches had progressed to the point that they were limiting her mobility. She denied fever, chills, chest pain, palpitations, shortness of breath, skin rashes, or joint pain. She denied recent illness, trauma, physical exertion, excessive heat exposure, or use of medications. The patient had been sluggish and was gaining weight over the past 2 months but she denied cold intolerance, menstrual abnormalities, or peripheral edema. One month ago, she developed a 2-3 cm nodule in her anterior neck that spontaneously resolved within a few days.

On initial examination her vital signs were temperature of 36.8°C, pulse rate of 90/minute, respiratory rate of 16/minute, blood pressure of 116/66 mm Hg, and oxygen saturation of 99%. Her body mass index was 23.03 kg/m^2^. Physical examination revealed a female in mild distress due to muscle pain. Head and neck exam was normal with no thyroid or anterior neck swelling. No lymphadenopathy was appreciated. Her cardiac, pulmonary, and abdominal exams were unremarkable. Exam of the extremities revealed diffuse muscle tenderness with limited flexion/extension and abduction/adduction of the upper and lower extremities due to bilateral pain. She had no peripheral edema.

A complete blood count showed a white blood cell count of 5,700/*μ*L with 13.7% eosinophils (780/*μ*L) and hemoglobin of 12.8 g/dL. A complete metabolic panel revealed normal sodium, potassium, and creatinine with an elevated AST level of 1248 units/L and ALT level of 481 units/L. Alkaline phosphatase and bilirubin were within normal limits. Creatinine phosphokinase (CPK) was 14,913 units/L (26–192 units/L), C-reactive protein was 1.69 mg/dL (0.000–0.300 mg/dL), and erythrocyte sedimentation rate was 9 mm/hr (0–12 mm/hr). An initial urinalysis showed large blood and 15–25 RBCs/HPF but the urine sample was contaminated with menstrual blood. Urine toxicology screen was negative. L-tryptophan ingestion was considered but the patient denied use of any supplements. Toxic oil syndrome was considered unlikely as she denied recent travel history or unusual food ingestion. We started treatment for rhabdomyolysis with aggressive intravenous fluid hydration. A liver ultrasound was negative for cirrhosis, biliary obstruction, or gallstones. Over the next 2 weeks, her CPK continued to increase to levels greater than 100,000 units/L despite aggressive hydration with intravenous fluids at rates of up to 300cc per hour. Bicarbonate was also added to maintain alkalinized urine. Her renal function remained stable with a BUN ranging from 3 to 13 mg/dL and creatinine ranging from 0.15 to 0.43 mg/dL. A repeat urinalysis at this time showed large blood, 0–2 RBCs/HPF, protein, and amorphous sediment.

Further diagnostic workup to elucidate the cause of rhabdomyolysis was unrevealing, with normal levels of thyroid-stimulating hormone, cortisol, aldolase, ANA, C-ANCA, P-ANCA, anti-Jo-1, anti-Ro, anti-La, anti-Sm, anti-RNP, dsDNA, and C3 and C4. Cardiac echogram and troponins revealed no cardiac involvement. Tests for infective causes including viruses (coxsackie, CMV, EBV, viral hepatitis, HSV, HIV, and influenza) and parasites (*Schistosoma*,* Giardia*,* Toxocara*,* Trichinella*, and* Strongyloides*) were negative, with the exception of influenza A and B antibody titers, which were 1 : 64 and 1 : 8, respectively. However, she did not report any upper respiratory tract symptoms that would have suggested influenza. A muscle biopsy was attained, as the etiology for rhabdomyolysis remained unclear. While awaiting the pathology report, she was started on steroids 10 days after admission for suspected inflammatory myopathy leading to a gradual improvement in her symptoms, CPK level, liver function tests, and peripheral eosinophilia.

**Figure 1:**
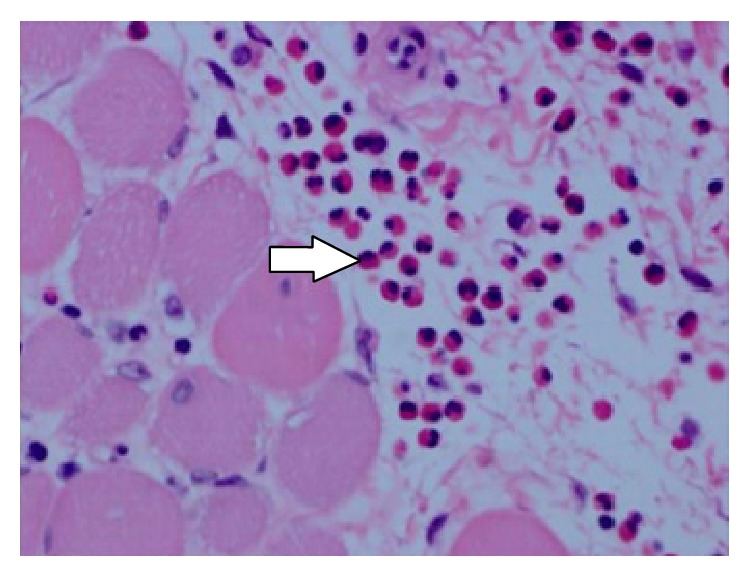
Cross section of skeletal muscle biopsy using H&E stain showing generalized myofiber atrophy with eosinophilic infiltrates (arrow) in the endomysium.

Her muscle biopsy demonstrated widespread eosinophilic infiltrate consistent with eosinophilic polymyositis as well as numerous plasma cells (Figure 1). There were no parasites like* Toxoplasma* or* Trichinella* seen on H&E stain. There were no hydatid cysts seen on biopsy making* Echinococcus* unlikely. While* Toxoplasma* and* Taenia solium* were not specifically tested for by serology, our patient did not display gastrointestinal or neurological symptoms to support these pathologies. SPEP and UPEP were normal making multiple myeloma unlikely. We did not suspect a hematological malignancy warranting a bone marrow biopsy. This biopsy result was not consistent with rhabdomyolysis caused by influenza based on review of the literature. Given significant improvement with steroids, this treatment was continued and the patient was advised to follow up with a neuromuscular disease specialist. She was seen in our internal medicine clinic after discharge and a slow steroid taper was continued for 6 months with successful remission of symptoms and normalization of CPK and liver enzyme levels, which would not be expected if an infectious entity were the cause.

## 3. Discussion

The present case illustrates an unusual cause of rhabdomyolysis. The etiologies of rhabdomyolysis are subdivided into four categories: exertional, nontraumatic exertional normal muscle, nontraumatic exertional abnormal muscle, and nonexertional. Our patient had an inflammatory myopathy, which is a nonexertional subtype. The inflammatory myopathies can be further divided into the rare eosinophilic myopathies (EM) and the more common noneosinophilic myopathies (NEM) like noneosinophilic polymyositis, dermatomyositis, and inclusion body myositis [19]. There are different classification systems to help diagnose these inflammatory myopathies but without biopsy and positive autoantibodies, identification remains a challenge [20].

Eosinophilic myositis (EM) usually presents between the ages of 14 and 70 and is twice as common in females compared to males [19]. The most common presenting symptoms include a gradual onset of muscle pain, edema of the upper and/or lower extremities, muscle weakness, and joint pains [19]. Other signs, symptoms, and lab findings of EM are listed in Tables 1 and 2. This slowly progressive myopathy mostly causes proximal muscle weakness with a marked increase in creatinine kinase.

Myositis with eosinophilic infiltrates most commonly involves parasites [17, 21] (*Trichinella*,* Echinococcus*,* Taenia solium*, and* Toxoplasma gondii*), viruses (EBV and coxsackie), inflammatory myopathies (dermatomyositis, polymyositis), and systemic diseases (Churg-Strauss syndrome) [13]. Other less common etiologies like muscular dystrophies (calpainopathy [8] and Becker Disease [14]), toxic exposures to L-tryptophan [7], toxic oil syndrome, malignancy, and EM as a component of idiopathic hypereosinophilic syndrome (HES) can also have eosinophilic predominant myositis [13]. Other drugs associated with myopathy and eosinophilia include cimetidine, phenytoin, and penicillamine [19]. Once all the above etiologies have been considered, and no cause has been identified, idiopathic eosinophilic myositis can be diagnosed as in our case.

Eosinophilia associated myopathy is categorized into 3 subtypes: focal eosinophilic myositis, eosinophilic perimyositis, and eosinophilic polymyositis (Table 3). Focal EM usually causes lower extremity pain and calf swelling. Eosinophilic perimyositis generally causes myalgias and mild proximal muscle weakness. Labs may show normal creatinine kinase levels. Eosinophilic polymyositis is more commonly a systemic disease with frequent cardiac, lung, or gut involvement [6, 13]. Interestingly, peripheral eosinophilia is not needed to diagnose any of the above entities [2, 15]. Clinically, our patient had severe muscle weakness, elevated CPK levels, a high degree of peripheral eosinophilia, and the need for steroids for symptomatic involvement. Histologically, her muscle biopsy revealed widespread eosinophilic infiltration consistent with a diagnosis of eosinophilic polymyositis.

The overall prognosis of EM is good and is most favorable in the localized form. As is shown in Table 2, eosinophilic polymyositis is the only subtype of EM that almost always requires prednisone for symptomatic improvement. However, the role of disease modifying drugs in eosinophilic polymyositis is yet to be determined. In some cases, IVIG [3, 10] and azathioprine have led to successful remission of the disease [3].

To the best of our knowledge, only two other cases of idiopathic eosinophilic polymyositis have been described in the English literature. In 1992, Behari et al. [2] described the case of a 24-year-old male who had muscle pain that gradually progressed for 2.5 years prior to presentation. Two months after treatment with steroids, his CPK levels remained elevated. In 1994, Mancias et al. [10] reported the case of an 8-year-old girl who had muscle weakness that progressed to myalgias. She also had an asthma exacerbation a few months prior to presentation. They initiated treatment with steroids but the patient's CPK levels remained elevated. They attempted intravenous immunoglobulins but a repeat muscle biopsy showed persistent eosinophilic infiltrate. However, no systemic involvement was noted in either case, including ours. Our patient presented with an acute onset of muscle pain with no prior complaints of weakness or myalgias, in contrast to the more insidious course described in the above two cases. Additionally, our patient responded well to 2 months of steroid therapy, with CPK levels returning to normal. Six months later her CPK levels remain within normal limits on a long steroid taper.

## 4. Conclusion

Myositis with eosinophilic infiltrates has a broad differential. This case report introduces an unusual presentation of an unusual illness and illustrates a systematic workup for rhabdomyolysis in the presence of peripheral eosinophilia. Before a diagnosis of eosinophilic myositis can be made, a wide array of diagnostic tests has to be completed. In order to determine the best treatment for a patient with EM, further defining the extent of eosinophilic infiltrate by a biopsy is of utmost importance. This case of idiopathic eosinophilic polymyositis, to our knowledge, is unique because it is a rare cause of severe rhabdomyolysis without another organ involvement. Clinicians should maintain a high index of suspicion for this physically debilitating disease to aid in prompt diagnosis.

## Figures and Tables

**Table 1:** Signs and symptoms of eosinophilic myositis [18].

Clinical features	Percentage
Muscle pain, cramping, or tenderness	68%
Upper or lower extremity swelling edema	45%
Muscle weakness	16%
Arthralgias/arthritis	10%
Myocarditis/pericarditis	10%
Vasculitis	6%
Inflammatory eye disease	6%
Raynaud's phenomenon	6%
Eosinophilic pneumonia	3%
Angioedema	3%

^*∗*^Permission for reuse in a journal was acquired from Elsevier.

**Table 2:** Laboratory findings of eosinophilic myositis [18].

Laboratory findings	Percentage
Peripheral eosinophilia (eosinophil count >4.5 × 10^8^)	77%
Inflammatory markers	
Elevated ESR	77%
Muscle markers	
Elevated CPK	68%
Elevated aldolase	44%
Autoimmune markers	
Rheumatoid factor	33%
ANA	6%

^*∗*^Permission for reuse in a journal was acquired from Elsevier. Table formatting was modified for clarification of content.

**Table 3:** Proposed criteria for diagnosis for eosinophilic myositis [13].

	Focal eosinophilic myositis^a^	Eosinophilic polymyositis^b^	Eosinophilic perimyositis^c^
Major	(1) Pain and calf swelling (other muscles can be affected) (2) Deep eosinophilic infiltration with muscle fiber invasion and necrosis on muscle biopsy	(1) Proximal weakness affecting limb girdle muscles (may be severe) (2) Widespread deep infiltration of eosinophil into muscles, with eosinophilic cuffing, on histology. Myonecrosis and endomysium inflammation usually +ve. If −ve deposition of MBP should be demonstrated by immunostain	(1) Myalgia, proximal mild weakness (2) Eosinophilic infiltrate confined to fascia and superficial perimysium, absence of myofiber necrosis

Minor	(1) ↑ CPK and aldolase (2) MRI or EMG evidence of focal myositis (3) Absence of systemic illness (4) Eosinophilia >0.5 × 10^9^/L	(1) ↑ CPK and aldolase (2) Eosinophilia >0.5 × 10^9^/L (3) Systemic illness with frequent cardiac involvement (4) Steroids are needed	(1) Absence of systemic manifestations (2) Normal CK and aldolase levels (3) Eosinophilia >0.5 × 10^9^/L

Exclude	DVT, cellulitis, parasitic infection	HES, cell T clonality, DM, vasculitis (CSS), drugs, calpainopathy, parasitic infections	Toxic oil syndrome, myalgia-eosinophilia, exposure to inorganic or organic substances

Treatment	No steroid treatment required. Symptoms resolve spontaneously	Prednisone 0.5–1 mg/kg/day is the treatment of choice	Rarely requires steroid treatment for symptom resolution

^a^2 major or 1 major and 3 minor criteria establish the diagnosis.

^b^Both major criteria or one major and two minor criteria establish the diagnosis.

^c^Both major criteria and major criteria number 2 plus two minor criteria enable the diagnosis.

^*∗*^Permission for reuse in a journal was acquired from Elsevier. The treatment section is an addition to the original table.

## Abbreviations

AST: Aspartate aminotransferase
ALT: Alanine aminotransferase
HPF: High powered field
ANA: Antinuclear antibody
C-ANCA: Cytoplasmic anti-neutrophil cytoplasmic antibody
P-ANCA: Perinuclear anti-neutrophil cytoplasmic antibody
Anti-dsDNA: Anti-double stranded DNA
Anti-Sm Ab: Anti-Smith antibodies
C3: Complement 3
C4: Complement 4
CMV: Cytomegalovirus
EBV: Epstein-Barr virus
HSV: Herpes simplex virus
HIV: Human immunodeficiency virus
H&E: Hematoxylin and eosin
SPEP: Serum electrophoresis
UPEP: Urine electrophoresis.
